# Oviposition by *Plagiodera versicolora* on *Salix matsudana* cv. ‘Zhuliu’ alters the leaf transcriptome and impairs larval performance

**DOI:** 10.3389/fpls.2023.1226641

**Published:** 2023-07-19

**Authors:** Fengjie Liu, Bin Li, Chenghu Liu, Yipeng Liu, Xiaolong Liu, Min Lu

**Affiliations:** State Key Laboratory of Biocatalysis and Enzyme Engineering, School of Life Sciences, Hubei University, Wuhan, China

**Keywords:** oviposition, RNA sequencing, differentially expressed genes, bioassays, *Plagiodera versicolora*, *Salix matsudana* cv. Zhuliu

## Abstract

Insect egg deposition can induce plant defenses against their larvae. Previous studies have primarily focused on herbaceous plant defenses; however, little is known about how the *Salicaceae* respond to insect egg deposition and defend themselves against herbivores. By combining plant defense gene studies and bioassays, we investigated the effect of the coleoptera *Plagiodera versicolora* egg deposition on willow (*Salix matsudana* cv. ‘Zhuliu’) and examined the interactions at the plant resistance and transcriptome levels. RNA-seq data were utilized to analyze changes in the leaf transcriptome with and without oviposition, and also the changes in the leaf transcriptome of feeding-damaged leaves with and without prior oviposition. *P. versicolora* oviposition on willow leaves resulted in altered expression levels of transcripts associated with plant stress and metabolic responses. Compared with leaves with no oviposition, leaves with egg deposition showed a slight increase in phenylpropanoid biosynthesis and phytohormone signaling genes after larval feeding. The RNA-seq analysis revealed alterations in willow transcripts in response to leaf beetle infestations. Bioassays indicated that oviposition by *P. versicolora* on willows reduced subsequent larvae performance, suggesting that prior oviposition by *P. versicolora* could increase willows’ resistance to larvae. This study advances our knowledge of how oviposition by coleoptera insects induces changes in the resistance of leaves to herbivory in the *Salicaceae* family.

## 1 Introduction

Trees dominate terrestrial ecosystems and provide habitats for many insects (Basset et al., 2012). Over time, trees have evolved various mechanisms to defend themselves against herbivores (Eyles et al., 2010; Caldwell et al., 2016). Multiple studies have demonstrated that plants are capable of responding to imminent stress cues, enhancing their induced stress resistance, and preparing them for potential damage (Mauch-Mani et al., 2017). These cues include insect feeding, leaf volatile emissions by damaged neighboring plants (Heil and Silva Bueno, 2007; Pashalidou et al., 2020), insect sex pheromones (Bittner et al., 2019), and insect egg deposition (Hilker and Fatouros, 2016).

Insect egg deposition triggers plant defense mechanisms that can not only directly affect egg survival but also indirectly increase the defense response against their larvae (Hilker and Fatouros, 2015; Hilker and Fatouros, 2016). Plant defense strategies induced by egg deposition specifically target the eggs themselves rather than the ovipositing female. These strategies include plant-mediated egg desiccation, dropping, crushing, and killing (Yamasaki et al., 2003; Desurmont and Weston, 2011; Petzold-Maxwell et al., 2011). In addition, plants can utilize egg deposition as a reliable signal to predict and prepare for a subsequent larval attack. Prior egg deposition can induce alterations in the quality of feeding-damaged leaves, resulting in impaired larval performance (Pashalidou et al., 2013; Austel et al., 2016; Bandoly et al., 2016; Bertea et al., 2020). For example, when *Spodoptera exigua* fed on *Nicotiana attenuata* leaves with prior egg deposition, larvae suffered higher mortality than those that fed on plants without prior egg deposition (Bandoly et al., 2015).

Molecular analyses revealed that plants exhibit substantial transcriptome changes in response to oviposition cues (Reymond, 2013; Bonnet et al., 2017; Ojeda-Martinez et al., 2022). Several studies have demonstrated that oviposition induces the expression of various defense-related genes in plants. These genes encompass those encoding pathogenesis-related (PR) proteins, responding to biotic and abiotic stresses, regulators of cell death, and innate immunity, and also stressors associated with the production of secondary metabolites, among other things (Little et al., 2007; Büchel et al., 2012). Furthermore, plants with prior oviposition showed stronger defense gene expressions after larval feeding, such as *Arabidopsis*, tobacco, tomato, and elm (Kim et al., 2012; Bandoly et al., 2016; Altmann et al., 2018; Lortzing et al., 2020). Moreover, several studies have shown that the increased efficiency of defense against larvae caused by oviposition is associated with changes in phytohormone levels. For instance, *S. exigua* larvae feeding on *N. attenuata* leaves with prior oviposition could increase the proteinase inhibitor activity in plants (Bandoly et al., 2015). Similarly, when the elm beetle *Xanthogaleruca luteola* fed on elm leaves with prior egg deposition, the plant was able to increase leaf gene expression levels of phenylpropanoid derivatives (Lortzing et al., 2019).

*Plagiodera versicolora* Laicharting (Coleoptera, Chrysomelidae) is a worldwide forest pest (Utsumi et al., 2009) whose larval and adult stages feed primarily on *Salicaceae* trees such as willows and poplars (Li et al., 2022). Female *P. versicolora* lay eggs almost daily while feeding on young leaves (Ma et al., 2021). The eggs are neatly arranged, usually sticking vertically to the back of the leaf. Studies have shown that crude extracts obtained from the surface of *P. versicolora* egg masses using organic solvents can attract females to lay eggs, while aqueous extracts have been found to repel females (Yang et al., 2005). Willow is a widespread tree throughout the world and is consumed extensively by various herbivores (Tahvanainen et al., 1985; Hallgren, 2003). Recent studies have shown that the egg deposition of *Nematus oligospilus* Förster (Hymenoptera, Tenthredinidae) on willow could increase the plant’s jasmonic acid levels, alter its volatile profile, and reduce neonate larval growth (Dávila et al., 2023). However, the effect of coleoptera egg deposition on willow trees and newborn larvae has not been reported. Hence, we used bamboo willow (*Salix matsudana* cv. ‘Zhuliu’) and *P. versicolora* as a model, and used *de novo* assembled RNA-seq data analysis and bioassays to investigate the willow trees’ response to *P. versicolora* egg deposition and larvae. Our results showed that oviposition by *P. versicolora* on willow alters the leaf transcriptome and impairs larval performance.

## 2 Materials and methods

### 2.1 Plants and insects

One-year-old bamboo willows (*Salix matsudana* cv. ‘Zhuliu’) were purchased from Xuanyu Flower Garden, Wuhan, China. The trees were planted in 7-L pots with a 3:1:1 mix of nutrient soil, pearlite, and vermiculite and transferred to a greenhouse (26°C ± 1°C, 60% ± 5% Relative Humidity (RH), Light 16h: Dark 8h (L16:D8). The willow plants were grown for a period of 6 weeks before they were used for the experiments.

*P. versicolora* adults were collected from the surrounding Sha Lake Park in Hubei Province, Wuhan, China, and reared in ventilated plastic boxes measuring 20 cm × 10 cm × 8 cm, in which they were fed fresh willow leaves. The rearing conditions were maintained in a greenhouse (26°C ± 1°C, 60% ± 5% RH, L16:D8).

### 2.2 Plant treatments

For all experiments conducted, willows of the same genotype and similar size were carefully selected to ensure that the plants used in each replicate block were comparable. In the experiment, the willows were subjected to the following treatments: (i) *P. versicolora* egg deposition (E), (ii) feeding-damaged leaves without prior egg deposition (CF), (iii) feeding-damaged leaves with prior egg deposition (EF), and (iv) control (C). Experiments were conducted using intact leaves (attached to the plants). Three replicate plants were harvested for each time point and treatment.

To obtain egg deposition plants (E), mated *P. versicolora* females were used to lay eggs on the surface of the willow leaves. After 3 days, when the eggs were about to hatch, we carefully removed the eggs from the leaves with forceps, and the leaves were collected immediately. In the feeding-damaged leaves treatments (CF and EF), the leaves were exposed to a specific number of newly hatched larvae. In this case, 10 newly hatched larvae were placed on feeding-damaged leaves of each plant. Larvae were removed after 24 h and leaf samples were collected.

### 2.3 RNA extraction, cDNA library construction, and Illumina sequencing

Approximately 100 mg of each leaf sample was collected and promptly frozen in liquid nitrogen for storage. Three biological replicates were used for each treatment or condition.

Total RNA was extracted using TRIzol reagent (Invitrogen, Carlsbad, CA, USA) following the manufacturer’s instructions. One percent agarose gels were used to visualize the integrity of the RNA samples and check for any signs of degradation or contamination. RNA integrity and purity were further assessed using the 2100 Bioanalyzer (Agilent Technologies). The concentration was quantified using the ND-2000 spectrophotometer (NanoDrop Technologies).

Approximately 2 μg of total RNA per sample was used for cDNA library construction. The cDNA libraries were prepared following Illumina® Stranded mRNA Prep, Ligation from Illumina (San Diego, CA), and then subjected to sequencing using the Illumina Novaseq 6000 platform. Sequencing was performed by Shanghai Majorbio Bio-pharm Technology Co., Ltd.

### 2.4 *De novo* assembly and gene annotation

Raw paired-end reads obtained from the sequencing were subjected to trimming and quality control using fastp (Chen et al., 2018), with default parameters. Clean data of the samples were then assembled *de novo* using Trinity (Grabherr et al., 2011). After assembly, they were further evaluated and optimized using BUSCO (Benchmarking Universal Single-Copy Orthologs) (Manni et al., 2021), TransRate (Smith-Unna et al., 2016), and CD-HIT (Fu et al., 2012). The assembled transcripts were searched again using the NR (NCBI protein non-redundant), Swiss-Prot, Pfam (Protein families), eggNOG (evolutionary genealogy of genes: Non-supervised Orthologous Groups), GO (Gene Ontology), and KEGG (Kyoto Encyclopedia of Genes and Genomes) databases using BLASTX to identify the proteins that had the highest sequence similarity. During the annotation process, a typical cut-off E-value of less than 1.0 × 10^−5^ was set to determine significant matches between the transcripts and the annotated proteins.

### 2.5 Differential expression analysis and functional enrichment

To calculate the transcript expression levels of differential expression genes (DEGs), the transcripts per million reads (TPM) method was employed. RNA-Seq by Expectation-Maximization(RSEM) (Li and Dewey, 2011) and Differential Expression analysis for Sequence Count data 2 (DESeq2) (Love et al., 2014) were used to quantify gene abundance and perform differential expression analysis, respectively. DEGs with |log2FC| ≥ 1 and False Discovery Rates (FDR) ≤ 0.05 (DESeq2) were considered as significantly different expressions.

Functional enrichment analyses including GO and KEGG were conducted to identify the significantly enriched GO terms and metabolic pathways among the DEGs. A Bonferroni-corrected *P*-value ≤ 0.05 was used to determine significant enrichment. Goatools and KOBAS were used for GO functional enrichment and KEGG pathway analysis, respectively (Xie et al., 2011).

### 2.6 Quantitative real-time PCR analysis

The cDNA was reverse transcribed using HiScript® III RT SuperMix for qPCR (Vazyme, Nanjing, China). The specific primers for qPCR analysis were provided in **Supplementary Table 1**. The reference gene used in this study was *ACT7* (Li et al., 2016). A qRT-PCR analysis was performed on the CFX Connect Real-Time System (Bio-Rad, Hercules, CA, USA). The ChamQTM Universal SYBR^®^ qRT-PCR Master Mix (Vazyme, Nanjing, China) was used for reactions, following the manufacturer’s instructions. The qRT-PCR reaction programs were as follows: 95°C for 30 s, 40 cycles of 95°C for 5 s, and 60°C for 30 s. Gene expression profiles were analyzed using the 2^−ΔΔCT^ method (Livak and Schmittgen, 2001); three replicates were used for each gene.

### 2.7 Insect performance

To count the number of eggs laid by *P. versicolora* and the hatching rate, only one mated female was allowed to oviposit on the leaves of each plant. A total of seven willows and seven mated females were used. The number of eggs on each leaf was counted at the end of oviposition by the female. After 3 days, the number of larvae hatched on the plants was recorded.

To clarify the effect of prior egg deposition on willows on *P. versicolora* larval growth, we measured larval performance after feeding on willow leaves with and without egg deposition. Using egg deposition and control plants, the eggs were removed after 3 days of oviposition. Ten newly hatched larvae were placed in the same position on the plant to allow the larvae to feed freely. Four replicates were used in each group. We compared the larval survival rate and body weight of willows with and without prior *P. versicolora* egg deposition. Larval mortality was recorded daily. Body weight was recorded every 2 days with an electronic balance (BT1251, Sartorius Scientific Instruments, China).

### 2.8 Statistical analysis

R software, version 4.2.2 (R Core Team, 2022), was used for all statistical analyses. Normal distribution and variance homogeneity were assessed using Shapiro–Wilk and Levene’s tests, respectively, or visually inspected through Q–Q plots.

We conducted an analysis to examine the impact of prior egg deposition on larval mortality in willows. Survival curves were compared using the logrank (Mantel–Cox) test. To compare the larval body weight between willows with and without prior *P. versicolora* egg deposition, linear mixed models (LMMs) were employed for the analysis. The LMMs included the replicate block as a random factor to account for potential variability.

For evaluating the qRT-PCR data and testing gene expression values, we utilized a one-way analysis of variance (ANOVA). Subsequently, Tukey’s HSD test was applied for *post-hoc* comparisons between different groups.

## 3 Results

### 3.1 RNA sequencing and *de novo* assembling of transcriptome

**Figure 1:**
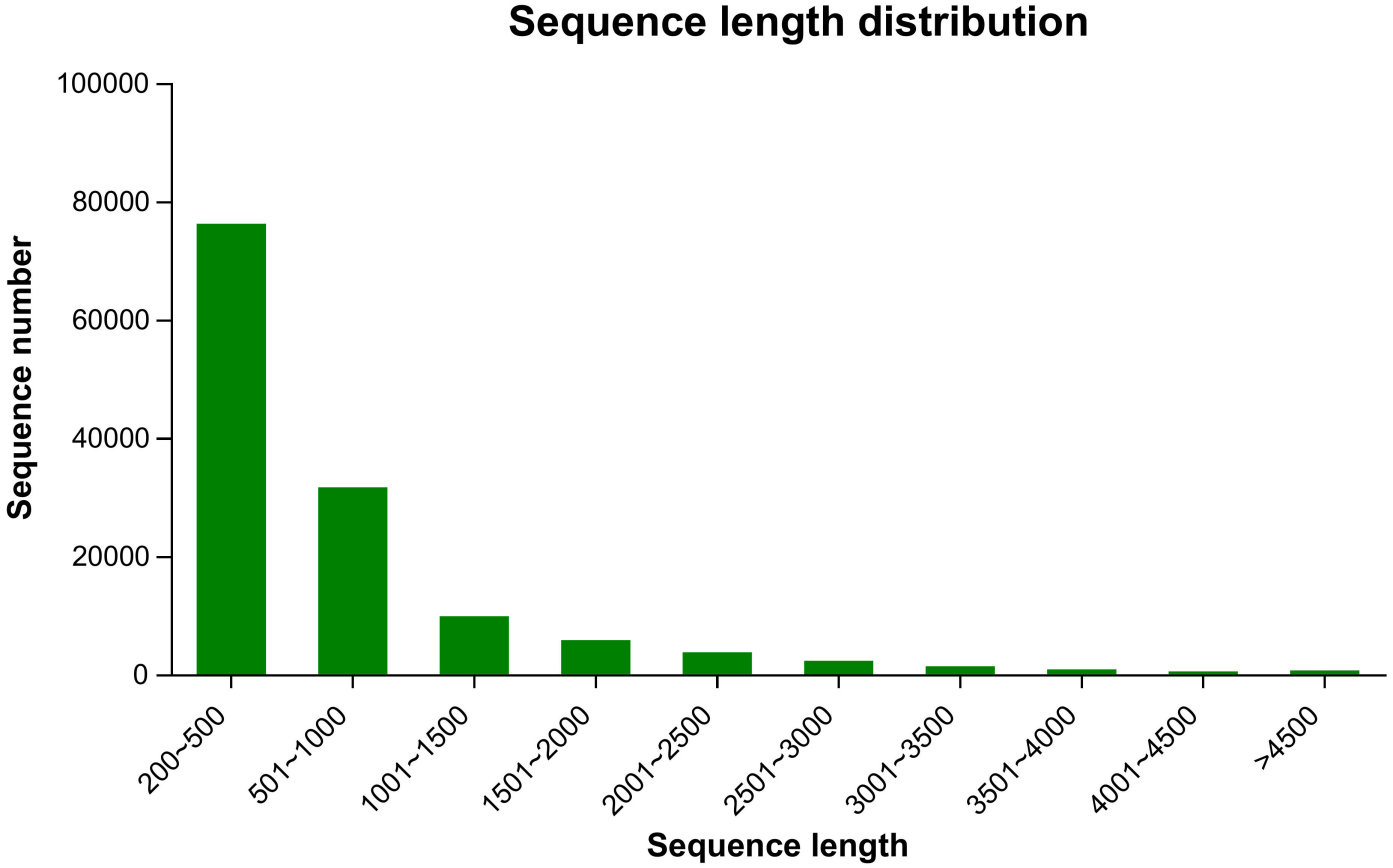
Length (bp) distribution of unigenes in the willow leaf transcriptome.

To investigate willow response to *P. versicolora* egg deposition and the subsequent larval feeding, we conducted transcriptome sequencing of leaves that had been subjected to various treatments. We obtained 110.47 Gb of raw sequence data, and 92.31% of the bases had a sequence quality score of >Q30. About 132,452 unigenes, with a total length of 95,157,991 and an N50 length of 1,050 bp, were identified (**Table 1**). Length distribution analysis showed that 42.47% (55,171) of all unigenes were longer than 500 bp in size (**Figure 1**).

**Table 1:** Result of the *de novo* transcriptome assembly performed with Trinity.

Type	Unigene	Transcript
Total sequence number	132,452	266,041
Total sequence base	95,157,991	230,466,949
Largest length	16,793	16,793
Smallest length	201	201
Average length	718.43	866.28
N50 length	1,050	1,323
E90N50 length	2,138	1,779
Percent GC	39.97	40.66

**Figure 2:**
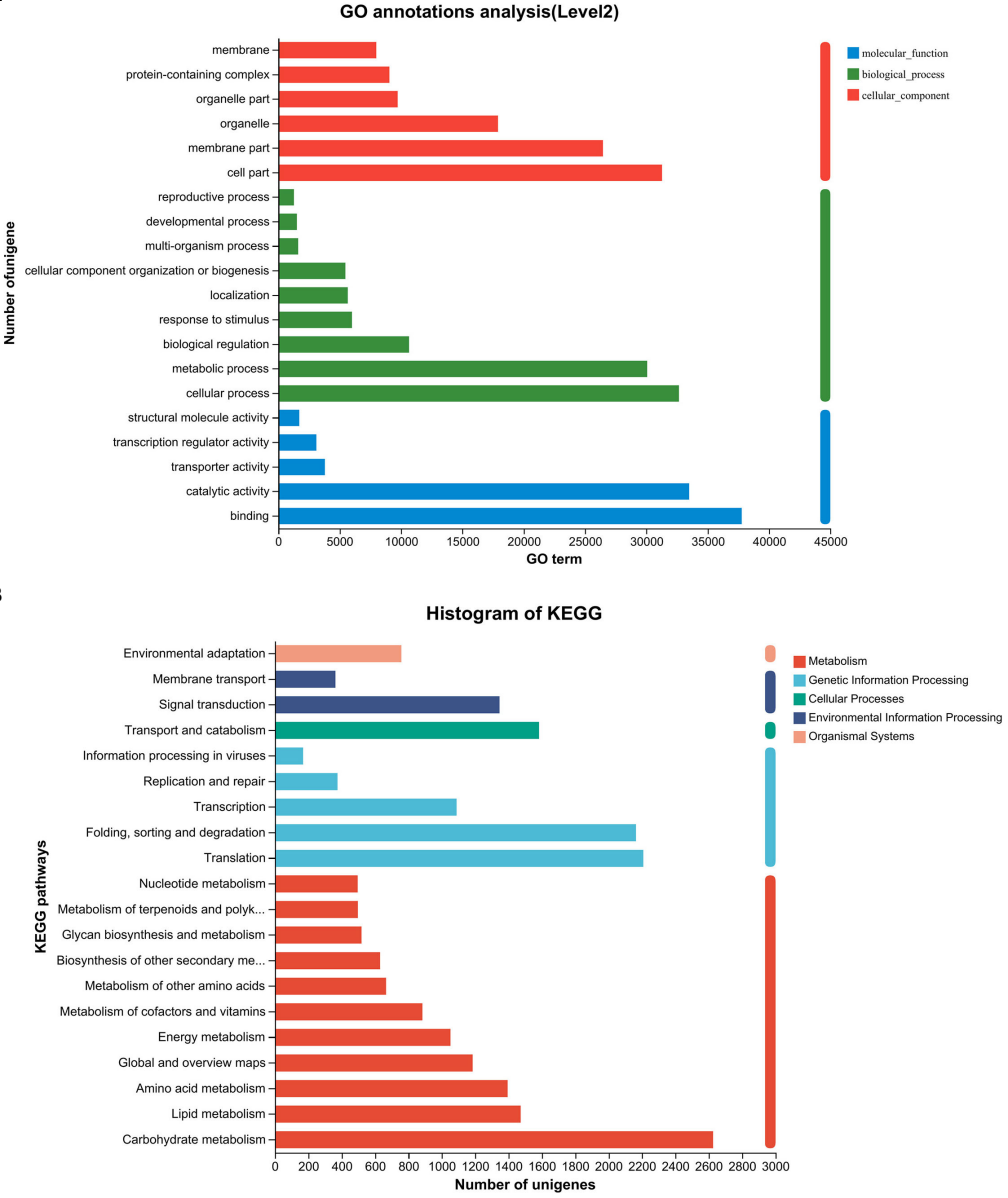
Gene function classification of unigenes in the willow leaf transcriptome. **(A)** Main GO categories of unigene. **(B)** KEGG metabolic pathway of unigenes.

Following the assembly of the transcriptome, annotations for the assembled unigenes were performed using BLAST in six public databases, namely NR, Swiss-Prot, Pfam, eggNOG, GO, and KEGG (**Table 2**). In the GO analysis, the unigenes were categorized into three main functional categories: biological process (96,417), cellular component (104,536), and molecular function (83,283) (**Figure 2A**). In the KEGG pathway database, the unigenes were classified into five main categories: the largest category was metabolism (11,423), followed by genetic information processing (6,005), environmental information processing (1,708), cellular processes (1,583), and organismal systems (758) (**Figure 2B**).

**Table 2:** Functional annotation of unigenes.

Database	Number of unigenes	Percentage
NR	82,476	62.50%
Swiss-prot	50,359	38.16%
Pfam	36,123	27.37%
eggNOG	70,967	53.78%
GO	69,142	52.39%
KEGG	32,361	24.52%
Total	85,676	64.92%

NR, NCBI protein non-redundant; evolutionary genealogy of genes: Non-supervised Orthologous Groups; GO, Gene Ontology; KEGG, Kyoto Encyclopedia of Genes and Genomes.

### 3.2 Transcriptional profiling of willow responses to *P. versicolora* egg deposition

**Figure 3:**
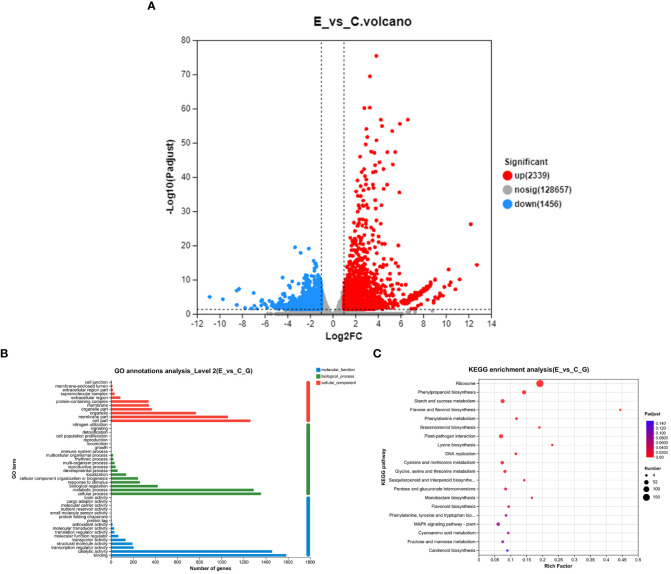
With and without prior *P. versicolora* egg deposition induces changes of DEGs (differential expression genes) in the transcriptome. **(A)** Volcano map analysis of DEGs. **(B)** GO annotation analysis of DEGs. **(C)** The top 20 enriched KEGG pathways of DEGs. G refers to unigene.

To assess the impact of *P. versicolora* oviposition on the transcription level of willow leaves, we compared the gene expression between leaves exposed to egg deposition (E) and untreated leaf samples (C) after a 3-day period (E vs. C). A total of 3,795 genes were significantly differentially expressed after *P. versicolora* egg deposition (2,239 up-regulated and 1,456 down-regulated) (**Figure 3A**).

In the GO functional analysis, the DEGs were successfully classified into 46 categories (E vs. C) (**Figure 3B**). Among these categories, the biological processes categories with the highest number of transcripts were “cellular and metabolic processes” and “biological regulation and stimulus-response” (**Figure 3B**). KEGG enrichment analysis was mapped to 118 KEGG pathways (**Figure 3C**). The pathways with the highest unigene representations were ribosome, followed by phenylpropanoid biosynthesis, and starch and sucrose metabolism (**Figure 3C**). These results suggest that the *P. versicolora* egg deposition could induce changes in the expression levels of transcripts associated with plant stress and metabolic responses.

### 3.3 Differentially expressed in feeding-damaged leaves with and without prior egg deposition

**Figure 4:**
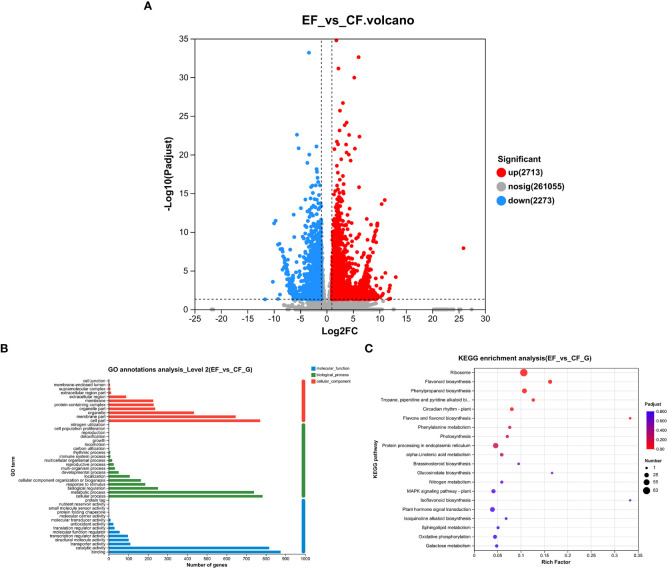
Feeding-damaged leaves with and without prior *P. versicolora* egg depositions showed induced changes in DEGs (differential expression genes) in the transcriptome. **(A)** Volcano map analysis of DEGs. **(B)** GO annotation analysis of DEGs. **(C)** The top 20 enriched KEGG pathways of DEGs. G refers to Unigene.

To study transcriptomic changes in willows induced by *P. versicolora* larval feeding on leaves with prior egg deposition, this treatment was compared with feeding-damaged leaves without prior egg deposition. Results indicate that in the feeding-damaged plants with egg deposition treatment (EF vs. CF) a total of 2,338 genes were significantly differentially expressed (1,385 up-regulated and 953 down-regulated) (**Figure 4A**).

In the GO functional analysis, the DEGs were successfully classified into 44 categories (EF vs. CF) (**Figure 4B**). Among these categories, the biological processes categories with the highest number of transcripts were “cellular and metabolic processes” and “biological regulation and stimulus-response” (**Figure 4B**). The KEGG enrichment analysis was mapped to 117 KEGG pathways (**Figure 4C**). The pathways with the highest unigene representation were ribosome, followed by flavonoid biosynthesis and phenylpropanoid biosynthesis (**Figure 4C**). Comparison of transcriptome changes in feeding-damaged leaves with and without prior *P. versicolora* egg deposition revealed EF group enrichment of transcripts associated with the plant secondary metabolism pathway, such as flavonoid biosynthesis, phenylpropanoid biosynthesis, Lavone and flavonol biosynthesis, and phenylalanine metabolism (**Figure 4C**). Thus, egg deposition of *P. versicolora* on willows potentially increases the regulation of plant defense responses to larvae.

### 3.4 qRT-PCR validation of the candidate genes

**Figure 5:**
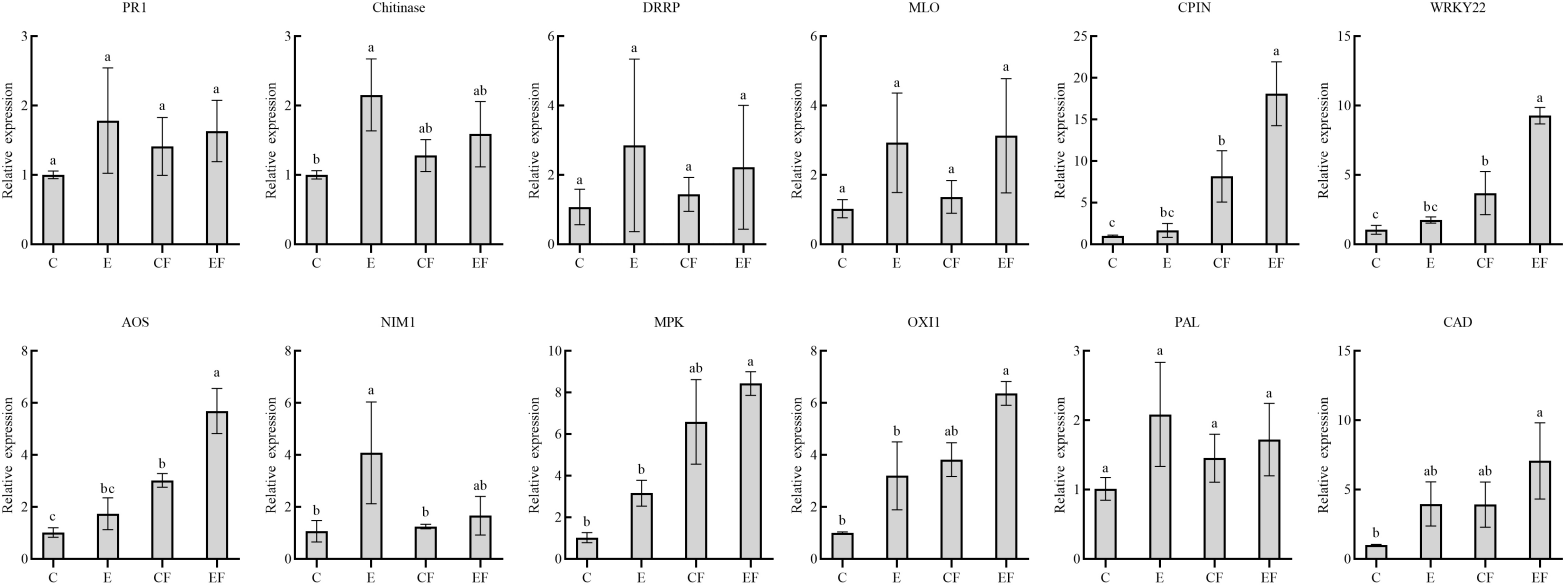
Expression analysis of 12 candidate genes in four treatments by qRT-PCR. (i) *P. versicolora* egg deposition (E), (ii) feeding-damaged leaves without prior egg deposition (CF), (iii) feeding-damaged leaves with prior egg deposition (EF), and (iv) control (C). The columns represent averages with vertical lines indicating standard error (SE). The differences in lowercase letters above each bar indicate significant differences (*P* < 0.05).

To analyze gene expression patterns associated with *P. versicolora* egg deposition, feeding-damaged leaves with and without prior egg deposition on leaves were validated by qRT-PCR. We selected 12 unigenes with high significance levels in GO and KEGG in the “response to wounding,” “defense response,” “hormone metabolic process,” and “Phenylpropanoid biosynthesis” categories. Compared with the control, the gene expression of pathogenesis-related protein 1 (PR 1), chitinases, disease resistance response protein (DRRP), and MLO-like protein increased after 3 days of egg deposition on plants (E), and there was a significant difference in chitinases. Cysteine proteinase inhibitor (CPIN) and the H2O2-related gene WRKY22 expression levels were significantly increased in leaves damaged by feeding with prior egg deposition (EF). The phytohormone salicylic acid (SA)-related gene NIM1 was significantly increased in egg deposition plants (E). Jasmonic acid (JA)-related gene AOS, ethylene-related genes mitogen-activated protein kinases (MAPKs), and oxidative signal inducible 1 (OXI1) significantly increased in EF plants. Phenylalanine ammonialyase (PAL) and cinnamyl alcohol dehydrogenase (CAD), which are related to the phenylpropanoid pathway, increased in plants treated with prior egg deposition (**Figure 5**).

### 3.5 *P. versicolora* egg deposition on willow could impair larvae performance

**Figure 6:**
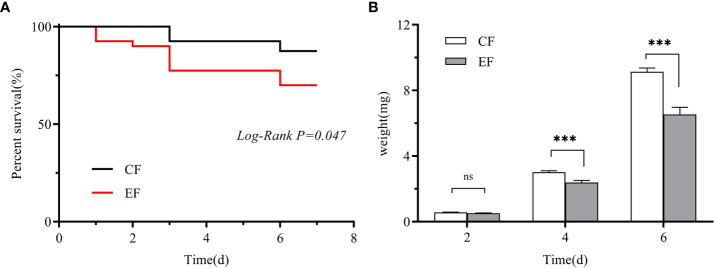
Performance of *P. versicolora* larvae feeding on leaves with and without prior egg deposition. CF: feeding-damaged leaves without prior egg deposition; EF: feeding-damaged leaves with prior egg deposition. **(A)** Survival curves for larvae feeding on plants with and without egg deposition. **(B)** Body weights of larvae at days 2, 4, and 6 on plants that were egg-free and plants with egg deposition, respectively. White bars indicate CF and gray bars indicate EF. Values are the means ± SE of four biological replicates. Asterisks indicate significant differences according to linear mixed models and significance levels: n.s: *P* > 0.05, ***: *P* < 0.001).

We measured the egg hatching rate and larval performance of *P. versicolora* on willows with prior egg deposition. The number of *P. versicolora* eggs on leaves was 22.14 ± 1.77, and the eggs hatched in about 3 days, with a hatching rate of 99.35%. Larval performance was measured in terms of survival and weight on willows with and without prior *P. versicolora* egg deposition. Larval mortality on leaves with prior egg deposition was significantly higher than on leaves without prior egg deposition (Mantel–Cox test, χ^2 ^= 3.94, *P*=0.047, **Figure 6A**). Similarly, the weight of larvae fed on leaves with prior egg deposition was lower than that of those fed on leaves without prior egg deposition (**Supplementary Table 2**). In particular, on the 4th and 6th days, the body weight was significantly reduced (**Figure 6B**; **Supplementary Table 2**). Overall, these results demonstrate the negative impact of *P. versicolora* egg deposition on willow leaves on the subsequent life stages of the larvae.

## 4 Discussion

### 4.1 *P. versicolora* egg deposition alters willow leaf transcriptome

This study was particularly focused on the expression of plant defense-related genes. Similarly, changes in defense-related genes were observed in the leaves of *Pieris brassicae* (Little et al., 2007) and *Xanthogaleruca luteola* (Büchel et al., 2012). In willows that were subjected to egg deposition, we observed gene expression levels increase, such as those of pathogenesis-related protein 1 (PR 1), chitinases, disease resistance response protein (DRRP), MLO-like protein, and NPR1/NIM1-interacting protein; this was verified by qRT-PCR, in which chitinases had significant differences. Our findings indicate that the expression of PR proteins by willows plays a potentially significant role in the response to *P. versicolora* egg deposition. PR proteins have been widely recognized for their involvement in defense responses following herbivore attacks (Van Loon et al., 2006). Chitinases, on the other hand, directly contribute to plant defenses by breaking down components of microbial cell walls (Veluthakkal and Dasgupta, 2010). In the case of *Arabidopsis thaliana*, chitinases are induced at and in the vicinity of the site where pierid eggs are laid, suggesting a potential defensive role against newly hatched larvae (Little et al., 2007). In *A. thaliana*, NPR1/NIM1 has been identified as a pivotal regulator of systemic acquired resistance (SAR) (Weigel et al., 2001). NPR1 governs the activation of PR genes involved in the synthesis of SA and plays a critical role in bridging the JA and SA signaling pathways (Spoel et al., 2003).

Oxidative signal inducible 1 (OXI1) plays a critical role in the signaling pathway that links oxidative burst signals to various downstream responses. OXI1 is necessary for the complete activation of MAPKs following exposure to reactive oxygen species (ROS) and elicitors (Rentel et al., 2004). Interestingly, our study demonstrated an elevation in the gene expression levels of both OXI1 and MAPKs in willow trees that were subjected to *P. versicolora* egg deposition (**Figure 5**). Previous research has indicated that the Hypersensitive Response (HR)-like response to pierid butterfly eggs on *A. thaliana* is associated with ROS accumulation, including hydrogen peroxide (Little et al., 2007; Bruessow et al., 2010; Gouhier-Darimont et al., 2013). However, *P. versicolora* egg deposition did not result in direct physical damage to the leaves, which is similar to the results of pierid butterflies laying eggs on *Arabidopsis*. Whether the deposition of *P. versicolora* eggs on willow leaves causes an HR-like response and what substances cause willow defense may be the focus of our future research.

Additionally, our observations show that the gene expression levels of the phenylalanine pathway are up-regulated in leaves of the willow plants with *P. versicolora* egg deposition. This suggests that insect oviposition could potentially increase the production of plant secondary metabolites, as has been documented in elms (Schott et al., 2021). Phenylalanine ammonia lyase (PAL) plays a critical role in initiating the phenylpropanoid pathway by catalyzing the deamination of phenylalanine (Tohge et al., 2017). The upregulation of this pathway and the activity of PAL highlight the involvement of phenylpropanoid metabolites in the defense response of willows against *P. versicolora* egg deposition. The exact phenylpropanoid-derived secondary metabolites will be studied in the future through metabolomics and HPLC analysis that has been successfully applied in other plant systems to uncover the composition and changing nature of their secondary metabolites.

### 4.2 *P. versicolora* egg deposition altered willow transcriptome response to larval feeding

Willow leaves damaged by *P. versicolora* feeding with prior egg deposition showed more transcript changes than leaves without egg deposition. This indicates that the initial egg deposition can prime the plant’s defense responses, leading to a more coordinated and effective defense against the subsequent feeding stages of the herbivore (Geuss et al., 2017).

Studies have shown that plant exposure to biotic or abiotic stress can influence their transcriptomic responses to subsequent stress (Conrath et al., 2015; Crisp et al., 2016). The transcriptional changes induced by insect eggs in plants can trigger an “alert” state, prompting the plants to reinforce their defenses against herbivores. Our results show that genes related to PR proteins and the cysteine proteinase inhibitor (CPIN) were also up-regulated. Additionally, the induction of lectins and protease inhibitors is known to possess anti-insect properties. Plant proteinase inhibitors have been shown to enhance defenses against insects and pathogens (Delaney et al., 1994). Studies have shown that anti-digestive proteins can impede the feeding of insects by inhibiting the activity of serine proteases in their digestive tracts (Mosolov and Valueva, 2005). *Tribolium castaneum* Herbst (Coleoptera, Tenebrionidae) guts usually show changes in digestive enzymes related to cysteine proteases and serine proteases when the larvae are treated with dietary cysteine peptidase inhibitors (Oppert et al., 2003; Oppert et al., 2010).

The enrichment of willow transcripts from GO categories associated with the phenylpropanoid pathway during larval feeding resulting from *P. versicolora* egg deposition suggests significant alterations in phenylpropanoid patterns. In tobacco leaves infested by moth larvae, prior egg deposition leads to enhanced levels of caffeoylputrescine (Bandoly et al., 2015); *A. thaliana* infested with butterfly eggs and larvae shows elevated levels of kaempferol derivatives (Lortzing et al., 2019); and elm exhibits enhanced phenylpropanoid transcriptional and metabolic responses to larvae (Schott et al., 2021). Thus, willow leaves that previously experienced *P. versicolora* egg deposition may be more effective in limiting herbivores due to the altered metabolite patterns induced by egg-mediated feeding. Using targeted and non-targeted metabolomics analyses and insect detoxification genes to explore the interaction between egg deposition-mediated phytochemical defenses and insects is important for future research.

### 4.3 Effects of egg deposition on insect performance

Our study shows that *P. versicolora* oviposition on willows could increase tree resistance to larvae. Specifically, when the *P. versicolora* larvae fed on plants with prior egg deposition, they experienced higher mortality and gained less weight (**Figure 6**). In general, these findings align with previous studies that have reported negative effects on the performance of herbivores when they feed on plants with prior egg deposition (Bandoly et al., 2015; Pashalidou et al., 2015). For example, the larvae of the pine sawfly *Diprion pini* (L.) experienced reduced weight gain and significantly higher mortality when they fed on pine twigs that had been previously laden with eggs (Beyaert et al., 2012).

However, these results were different from the effect of oviposition by a specialist hymenoptera, *Nematus oligospilus*, on *Salix babylonica* foliage (Dávila et al., 2023). Prior *N. oligospilus* egg depositions on *S. babylonica* did not have a significant impact on larval survival. While there was a slight difference in the average mass of mature larvae at 13 days between the egg-free treatment and the control foliage treatment, the difference was not statistically significant. In contrast, *N. oligospilus* experienced a significant increase in prepupal development time when feeding on foliage that had been previously subjected to egg deposition (Dávila et al., 2023). From current experiments, whether it is feeding-damaged leaves with or without prior *P. versicolora* egg deposition, they pupate in about 7 days. The effects of *P. versicolora* egg deposition were mainly concentrated in the larval stage, and the timing of pupae rearing may be similar to *X. luteola* and unaffected (Austel et al., 2016).

## 5 Conclusion

The present study confirms previous observations made in other plant species, demonstrating that oviposition by *P. versicolora* on willow (*Salix matsudana* cv. ‘Zhuliu’) increases the plant’s resistance to larvae. RNA-seq data analysis revealed changes in the transcriptome of willows with *P. versicolora* egg deposition. Similarly, there was a slight increase in the number of transcriptome changes in leaves with egg deposition after larval feeding damage compared with leaves without egg deposition. These findings suggest that willow not only has the ability to respond directly to *P. versicolora* egg deposition but can also enhance this response when encountering feeding larvae. Therefore, we provide evidence here that insect egg deposition causes transcriptome changes in plants and reduces larval performance, which will be important for further studies of other woody plants, deciduous trees, and insects.

## Data availability statement

The datasets presented in this study can be found in online repositories. The names of the repository/repositories and accession number(s) can be found below: https://www.ncbi.nlm.nih.gov/, PRJNA962771.

## Author contributions

FL and ML conceived and designed the experiment. FL, BL, and CL performed the experiment. FL and YL analyzed data and wrote the manuscript. XL and ML revised the manuscript. All authors contributed to the article and approved the submitted version.
