# Supplementary material for: Oviposition by Plagiodera versicolora on Salix matsudana cv. ‘Zhuliu’ alters the leaf transcriptome and impairs larval performance
Source: Front Plant Sci. 2023 Jul 19;14:1226641. doi: 10.3389/fpls.2023.1226641 (PMC10394651; doi:10.3389/fpls.2023.1226641)
Supplement: Supplementary file 1 [file Table_1.docx]

Supplementary Material

Oviposition by *Plagiodera versicolora* on willow alters the leaf transcriptome and impairs larval performance

Fengjie Liu^1^, Bin Li^1^, Chenghu Liu^1^, Yipeng Liu^1^, Xiaolong Liu^1^, Min Lu^1*^

*** Correspondence:** Min Lu [lumin@hubu.edu.cn](mailto:lumin@hubu.edu.cn)

# Supplementary Tables

**Supplementary Table 1.** List of primers used in qRT-PCR analysis.

| Gene | Unigene  (de novo assembly) | forward/reverse primer name | forward/reverse primer sequence (5'-3') |
| --- | --- | --- | --- |
| PR protein 1 | TRINITY_DN1104_c0_g1 | PR1_F | CATATGACCAGGGGACCACG |
|  |  | PR1_R | GCCGACCAAACGAAAATCCC |
| Chitinase | TRINITY_DN5762_c0_g1 | Chitinase_F | CTCTTCCGTGAATACATAGG |
|  |  | Chitinase_R | GAGGTGTCATAGTCAATGG |
| DRRP | TRINITY_DN31667_c0_g1 | DRRP_F | CCCATCACCTTAGACAAC |
|  |  | DRRP_R | CCCTTATGCTCAGTAGAG |
| MLO-like protein | TRINITY_DN593_c0_g1 | MLO_F | CAGCACTCAAAACCTGGCAC |
|  |  | MLO_R | ACTATCGGGAGCAGTGTTGC |
| WRKY22 | TRINITY_DN515_c0_g1 | WRKY22_F | TGGTTTTGGCAGTGACCAGG |
|  |  | WRKY22_R | TGGTGGCTTGCGGAGAAAAA |
| CPIN | TRINITY_DN14365_c0_g1 | CPIN_F | CTCCTCTTCATCGCTGTA |
|  |  | CPIN_R | GTCTTTCAAGTCCTCTATCG |
| AOS | TRINITY_DN1504_c0_g1 | AOS_F | CTACCGAGTTCTGTCCTA |
|  |  | AOS_R | CGTGACTTGAGGAGATAG |
| NIM1 | TRINITY_DN21217_c0_g1 | NIM1-F | CTGATGAATGCTCCAGATC |
|  |  | NIM1-R | CATCTCAGTCTCTTCCATG |
| MPK | TRINITY_DN7832_c0_g2 | MPK_F | GAGGAGCATTGTCAGTAC |
|  |  | MPK_R | CGCATATCTTCAGGTCAC |
| OXI1 | TRINITY_DN31941_c0_g1 | OXI1_F | CCTGAAGAATCCGATGAAG |
|  |  | OXI1_R | CTCCTGAACGAGAAAGAC |
| PAL | TRINITY_DN10323_c0_g1 | PLA_F | GAAGTAGAGAGTGCTAGAC |
|  |  | PLA_R | GACCTTCTCACCAGTAAG |
| CAD | TRINITY_DN12596_c1_g1 | CAD_F | CCATTGCAGTTCGTTACGCC |
|  |  | CAD_R | GTGGAGGCCAATCCGTTTTC |

**Supplementary Table 2.** Statistical models assessing the effect of oviposition on performance parameters of *Plagiodera versicolora* larvae kept on defined willow leaves. Summaries of linear mixed models (LMMs) testing the effect of oviposition on larval mass after (a) two days, (b) four days, (c) six days. Significant P-values are highlighted in bold.

| **(a) LMM fit by REML for larval mass on day 2** | | | | |
| --- | --- | --- | --- | --- |
| *Random effects* | **Variance** | **SD** |  |  |
| Replicate | 0.000 | 0.000 |  |  |
| *Fixed effects* | **Estimate** | **SE** | **Z** | ***P*** |
| Intercept | 0.565 | 0.016 | 35.557 | **< 0.001** |
| Eggs (yes/no) | -0.046 | 0.023 | -1.973 | 0.052 |
| **(b) LMM fit by REML for larval mass on day 4** | | | | |
| *Random effects* | **Variance** | **SD** |  |  |
| Replicate | 0.080 | 0.283 |  |  |
| *Fixed effects* | **Estimate** | **SE** | **Z** | ***P*** |
| Intercept | 2.994 | 0.171 | 17.550 | **< 0.001** |
| Eggs (yes/no) | -0.614 | 0.142 | -4.341 | **< 0.001** |
| **(c) LMM fit by REML for larval mass on day 6** | | | | |
| *Random effects* | **Variance** | **SD** |  |  |
| Replicate | 0.727 | 0.852 |  |  |
| *Fixed effects* | **Estimate** | **SE** | **Z** | ***P*** |
| Intercept | 9.115 | 0.5166 | 17.64 | **< 0.001** |
| Eggs (yes/no) | -2.571 | 0.43 | -5.93 | **< 0.001** |
